# Identifying and Analyzing Health-Related Themes in Disinformation Shared by Conservative and Liberal Russian Trolls on Twitter

**DOI:** 10.3390/ijerph18042159

**Published:** 2021-02-23

**Authors:** Amir Karami, Morgan Lundy, Frank Webb, Gabrielle Turner-McGrievy, Brooke W. McKeever, Robert McKeever

**Affiliations:** 1 School of Information Science, University of South Carolina, Columbia, SC 29208, USA; 2 School of Information Sciences, University of Illinois Urbana-Champaign, Champaign, IL 61820, USA; melundy2@illinois.edu; 3 Department of Computational and Data Sciences, George Mason University, Fairfax, VA 22030, USA; fwebb2@masonlive.gmu.edu; 4 Arnold School of Public Health, University of South Carolina, Columbia, SC 29208, USA; brie@sc.edu; 5 School of Journalism and Mass Communications, University of South Carolina, Columbia, SC 29208, USA; brookew@sc.edu (B.W.M.); robert.mckeever@sc.edu (R.M.)

**Keywords:** Twitter, trolls, health, disinformation, text mining, topic modeling

## Abstract

To combat health disinformation shared online, there is a need to identify and characterize the prevalence of topics shared by trolls managed by individuals to promote discord. The current literature is limited to a few health topics and dominated by vaccination. The goal of this study is to identify and analyze the breadth of health topics discussed by left (liberal) and right (conservative) Russian trolls on Twitter. We introduce an automated framework based on mixed methods including both computational and qualitative techniques. Results suggest that Russian trolls discussed 48 health-related topics, ranging from diet to abortion. Out of the 48 topics, there was a significant difference (*p*-value ≤ 0.004) between left and right trolls based on 17 topics. Hillary Clinton’s health during the 2016 election was the most popular topic for right trolls, who discussed this topic significantly more than left trolls. Mental health was the most popular topic for left trolls, who discussed this topic significantly more than right trolls. This study shows that health disinformation is a global public health threat on social media for a considerable number of health topics. This study can be beneficial for researchers who are interested in political disinformation and health monitoring, communication, and promotion on social media by showing health information shared by Russian trolls.

## 1. Introduction

Social media platforms such as Twitter have provided a great opportunity for millions of people to share their information [1]. While social media facilitates sharing truthful information, it also provides a platform to spread false information [2], which often spreads faster than true information [3]. Social media is the major source of misinformation and disinformation, and is in the front line of information warfare [4]. False information with and without intent to harm is misinformation and disinformation [4]. The most well-known form of disinformation has been called “fake news” [4].

Two traditional sources of health misinformation and disinformation are spams (e.g., advertisements [5]) and patients’ anecdotal experiences [6]. However, in recent years, social media has become a major source of health misinformation and disinformation [7,8]. In social media, most false health information is generated by automated accounts (bots) and individuals (trolls) “who misrepresent their identities with the intention of promoting discord” [9]. Seven-in-ten US adults believe that made-up information has significant harm to the nation [10], and eight-in-ten think that at least a fair amount of social media news comes from malicious actors [11].

Health misinformation and disinformation are a global public health threat [7]. False health information poses a negative impact on health communication and promotion and makes it difficult to find trustworthy information [12]. Health misinformation and disinformation have the potential to exert negative effects on public health, such as fostering hostility toward health workers during the Ebola outbreak, bolstering antivaccine movements, and eroding public trust in health systems and organizations [5]. False health information also leads to negative financial consequences, such as an estimated annual cost of $9 billion (USD) to the healthcare sector in the U.S. [13].

Among social media platforms, the prevalence of false information on Twitter is more than on Facebook and Reddit [12,14]. It was estimated that two-thirds of URLs shared on Twitter are posted by malicious actors [15]. On Twitter, different types of malicious actors, covering both automated accounts (including traditional spambots, social spambots, content polluters, and fake followers) and human users, mainly trolls, have been identified [14].

To combat false information, there is a need to identify and characterize the prevalence of topics shared by automated accounts. However, the current literature is limited to a few topics, dominated by vaccination [16]. Among state-sponsored datasets released by Twitter, Russia had the most social media activity related to information manipulation [17]. Russian trolls shared a wide range of political and nonpolitical topics between February 2012 and May 2018, with the vast majority during the 2016 election [17]. A research study used qualitative analysis to investigate categories of the trolls in a data sample. Two categories of trolls (left and right trolls) were identified with qualitative and quantitative analyses [18]. The left trolls expressed support for Bernie Sanders and opposed Hillary Clinton, and right trolls promoted the Donald Trump campaign [18]. Current research studying trolls focuses on three issues: (1) identifying and characterizing false information [3], (2) detecting trolls using different social media features such as emotional signals [19], topic and sentiment analyses [20], and social media activities and the content of social comments [21], (3) analyzing their political comments [22] and a few health topics such as vaccines [9], diet [22], the health of Hillary Clinton (Donald Trump’s competitor in the US 2016 election) [23], abortion [23], and food poisoning [23]. While current literature provides valuable insight, there is a need to address two important questions.

RQ1: What heath topics are present in tweets posted by left (liberal) and right (conservative) Russian trolls?

RQ2: Was there any difference between left and right Russian trolls based on the weight of health topics identified in RQ1?

To address these questions, we introduced an automated framework based on mixed methods including both computational and qualitative coding techniques. Current relevant studies either utilize qualitative methods that cannot be applied on large datasets or lack filtering methods to identify health-related tweets. Compared to the current research, our framework offered a new approach to identify health-related tweets and topics and compared the topics of left and right trolls. Our framework was based on utilizing multiple filtering methods using linguistics analysis and topic modeling, offering a systematic qualitative approach for topic analysis, and using statistical tests. The benefits of the proposed efficient approach rely on offering an automated flexible framework that can be adopted to not only large health datasets but also other massive datasets, such as political social comments.

This framework was applied to Russian trolls’ data including tweets from accounts associated with the Internet Research Agency (IRA), which is a Russian company that employs fake social media accounts to promote Russian business and political interests [24]. The IRA has interfered with US political processes by spreading disinformation via social media [25]. IRA was involved more on Twitter than any other social media site [26]. Twitter is a popular and powerful social media platform to amplify false health information. This social media platform has 300+ million active users in total, 500 million tweets per day, and 48+ million users in the US [27]. Our framework not only identified health topics but also compared left and right Russian trolls based on the average weight of the identified health topics. We believe this is a critical step to develop a successful strategy to fight false health information.

## 2. Materials and Methods

**Figure 1:**
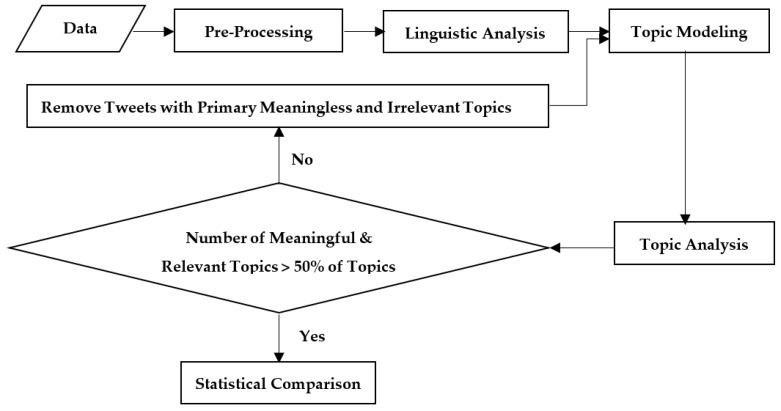
Research framework. This approach (i) cleaned and prepared data (e.g., removing short tweets), (ii) identified tweets containing health-related words (e.g., pain), (iii) applied topic modeling to disclose topics and their weight for each tweet, (iv) developed qualitative coding to analyze topics, (v) identified the primary topics of tweets and removed meaningless and irrelevant tweets, and (vi) utilized adjusted statistical tests to compare left and right trolls based on the weight of each topic per tweet. Steps iii, iv, and v were repeated until identifying more than 50% of meaningful and relevant topics.

The goal of this paper is to identify, analyze, and compare health comments shared by Russian trolls promoting left or right political ideologies. This section provides details on the data and methods used to address our research questions. We utilized mixed methods, including linguistic analysis, topic modeling, topic analysis, and statistical comparison to identify and compare health topics of left and right Russian trolls (Figure 1).

### 2.1. Data Pre-Processing

We obtained data from https://github.com/fivethirtyeight/russian-troll-tweets/(accessed on 3 October 2019). This data included around three million tweets posted between February 2012 and May 2018 from the IRA’s trolls divided into five categories, including Right Troll, Left Troll, News Feed, Hashtag Gamer, and Fearmonger, using a sequential mixed methods design [18]. The focus of this research was on tweets posted by the left and right trolls because they are the most important component of IRA [18]. For data preprocessing, we removed duplicate tweets and retweets, URLs in tweets, hashtags, usernames, and short-length tweets containing fewer than five words.

### 2.2. Identification and Analysis of Health Tweets and Topics

This process included two steps: linguistic analysis and topic modeling. We provide more details on utilizing and combining these two steps below.

#### 2.2.1. Linguistic Analysis

To identify health-related words, we used Linguistic Inquiry and Word Count (LIWC))( Pennebaker Conglomerates, Inc., Austin, TX, USA), which is a lexicon-based tool for text analysis with different categories [28]. LIWC has been utilized for different purposes, such as opinion mining [29] and spam detection [30], and supports popular languages, such as English and Spanish [28]. We used the health category of LIWC to identify tweets containing health words such as “pain”, “doctor”, and “calorie”, which provided a broad scope of health-related tweets.

#### 2.2.2. Topic Modeling

Some of the tweets contained health words but did not actually relate to health. For example, “she’s writing a book about her campaign and that it’s a painful process” contains “painful”, which is a health-related word, but does not discuss a health topic. To address this issue, we utilized topic modeling to find the topics of tweets containing health content and removed tweets representing nonhealth topics.

Among topic models, Latent Dirichlet Allocation (LDA) [31] is an effective model [32] that has been utilized for a wide range of domains, such as understanding public opinion [29,33], analysis of health documents [34,35], mining online reviews [36,37], and developing a systematic literature review [38,39,40]. LDA has also been used to characterize social media discussions on different issues, such as diet [41], exercise [42], LGBT health [43,44], antiquarantine discussions during the COVID-19 pandemic [45], politics [46], and natural disasters [47,48]. LDA creates topics including the probability of each word (W) given a topic (T) or P(W|T). LDA also represents the probability of each topic given a document (D) or P(T|D) [49]. In this study, each document represents a tweet. Therefore, for n tweets (documents), m words, and t topics, LDA builds two matrices:
Topics
Documents$\mathrm{Words}\left[\begin{array}{ccc} P(W_{1}|T_{1}) & \cdots & P(W_{1}|T_{t})\\ \vdots & \ddots & \vdots \\ P(W_{m}|T_{1}) & \cdots & P(W_{m}|T_{t}) \end{array}\right]$&$\mathrm{Topics}\left[\begin{array}{ccc} P(T_{1}|D_{1}) & \cdots & P(T_{t}|D_{n})\\ \vdots & \ddots & \vdots \\ P(T_{t}|D_{1}) & \cdots & P(T_{t}|D_{n}) \end{array}\right]$P(W_k_|T_k_)
P(T_k_|D_j_)

Within each topic, the top words based on the descending order of P(W_i_|T_k_) represent a topic that was then further interpreted by human coding, which we describe in the topic analysis section. P(T_k_|D_j_) helps find the primary topic of a tweet. The primary topic is the topic with the highest P(T_k_|D_j_) for a tweet. For example, suppose that there are three topics in a corpus and P(T_1_|D_1_), P(T_2_|D_1_), and P(T_3_|D_1_) are 0.1, 0.2, and 0.7, respectively. In this example, T3 is the primary topic of the first tweet (D_1_). After human coding, we removed tweets with primary topics that are meaningless and not related to health, e.g., astrology because of “cancer” or the television show Doctor Who due to its title. Then, we repeat this step until the majority (at least 51%) of topics are meaningful and health-related topics.

#### 2.2.3. Topic Analysis

To understand the topics generated by LDA, we utilized human coding (HC) with two coders. The two coders addressed the following binary (Yes/No) questions: (HC_Q1) Is the topic understandable? and (HC_Q2) Does the topic contain a health issue? Then, we conducted consensus coding [50] to address a short answer question for each meaningful and health-related topic. The third question was (HC_Q3) what is a proper label to represent the topic’s theme? In this step, the two coders developed an initial label for each meaningful and health-related topic independently using top words in each topic P(W_i_|T_k_) and reading the most relevant tweets using P(T_k_|D_j_) (see Appendix A for a tweet example for each topic offered by LDA). After achieving an acceptable agreement, the coders provided more details on their labels in a meeting to have standard labels. The coders could compare their labels after assigning the initial label and could change or keep their initial labels. A third coder resolved disagreements between the two coders. We used the percentage agreement to assess the reliability of coding.

### 2.3. Statistical Comparison

After achieving at least 51% meaningful and health-related topics, we developed statistical tests using the two-sample t-test developed in the R mosaic package [51] to compare left and right trolls based on the mean of P(T_k_|D_j_). The significance level was set based on sample size [52] using $\frac{0.05}{\sqrt{\frac{N}{100}}}$ [53], where N is the number of tweets. We also adjusted *p*-values using the False Discovery Rate (FDR) method [54] that minimizes both false positives and false negatives [55]. This step helped us find whether there was a significant difference between the left and right trolls based on the mean of P(T_k_|D_j_).

## 3. Results

We obtained 2,973,372 tweets posted by Russian bots on Twitter. Out of the collected tweets, 1,069,289 tweets were posted by left (405,549 tweets) and right (663,740 tweets) trolls. LIWC identified 55,734 tweets containing health-related words. For the first round of applying LDA, we estimated the optimum number of topics at 128 using C_V coherence analysis implemented in the gensim Python package [56]. Then, we applied the Mallet implementation of LDA [57] with the parameters of 128 topics and 4000 iterations. Out of the 128 topics, human coding identified 91 non-health or meaningless topics with 75.2% and 87.6% agreement on coding HC_Q1 and HC_Q2, respectively. This means that less than 50% of the 128 topics were meaningful and health-related topics, indicating that we did not achieve the 51% threshold. Therefore, we removed 40,486 tweets in which the primary topic was among those 91 topics. This process provided 15,249 tweets including 5837 and 9412 tweets posted by left and right trolls, respectively. These tweets contained 98,328 tokens (a string of contiguous characters between two spaces) and 9639 unique words.

**Figure 2:**
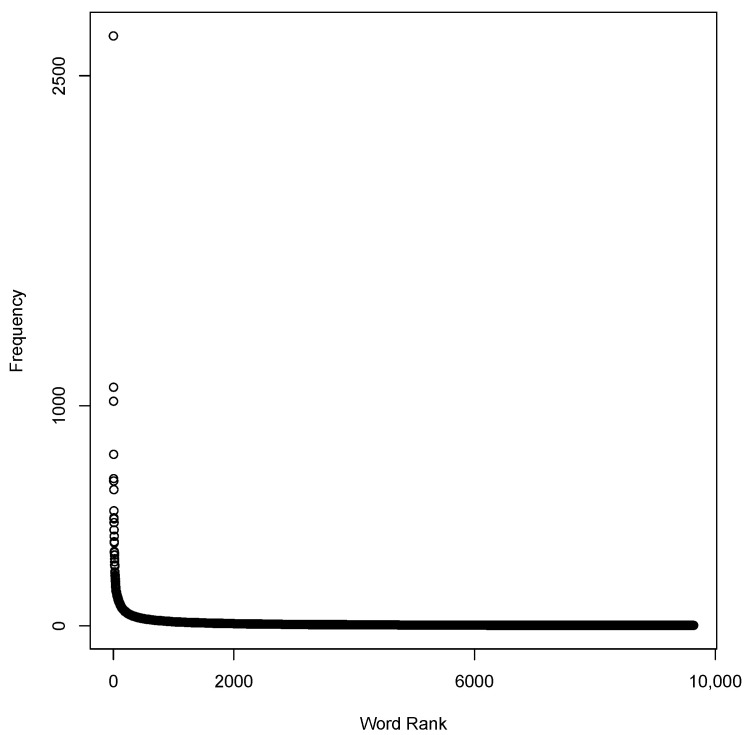
Frequency of words and Zipf’s law.

**Figure 3:**
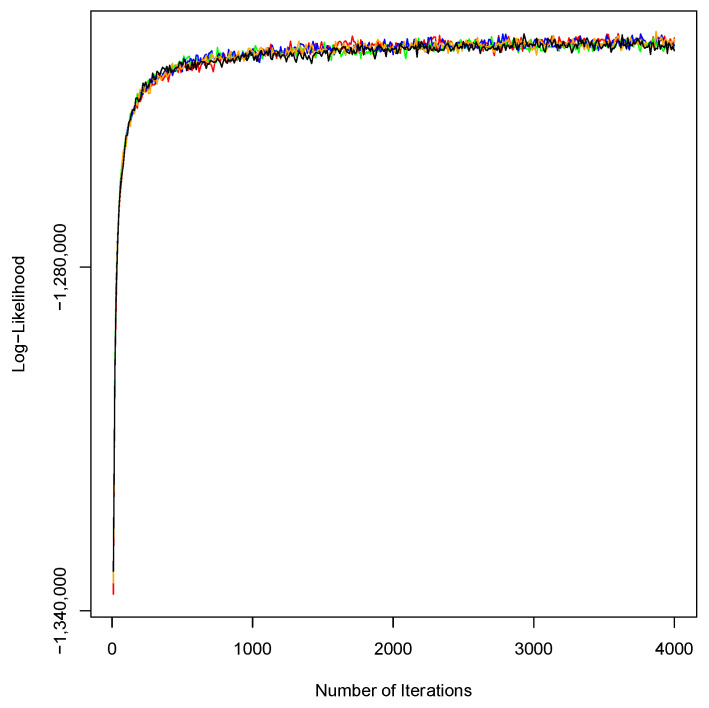
Convergence of the log-likelihood for five sets of 4000 iterations.

After estimating the number of topics at 78 using C_V coherence analysis, we applied LDA for the second round on the 15,249 tweets and achieved the 51% threshold by finding 48 (61%) meaningful and health-related topics labeled by the coders with 82.1%, 87.2%, and 88.9% agreement on HQ1–HQ3, respectively. The frequency of unique words was inversely proportional to its frequency rank (Figure 2), which was in line with Zipf’s law [58]. We investigated the robustness of LDA by comparing the mean of log-likelihood between five sets of 4000 iterations. The comparison illustrated that there was not a significant difference (*p*-value > 0.004) between the iterations, indicating a robust process (Figure 3).

**Figure 4:**
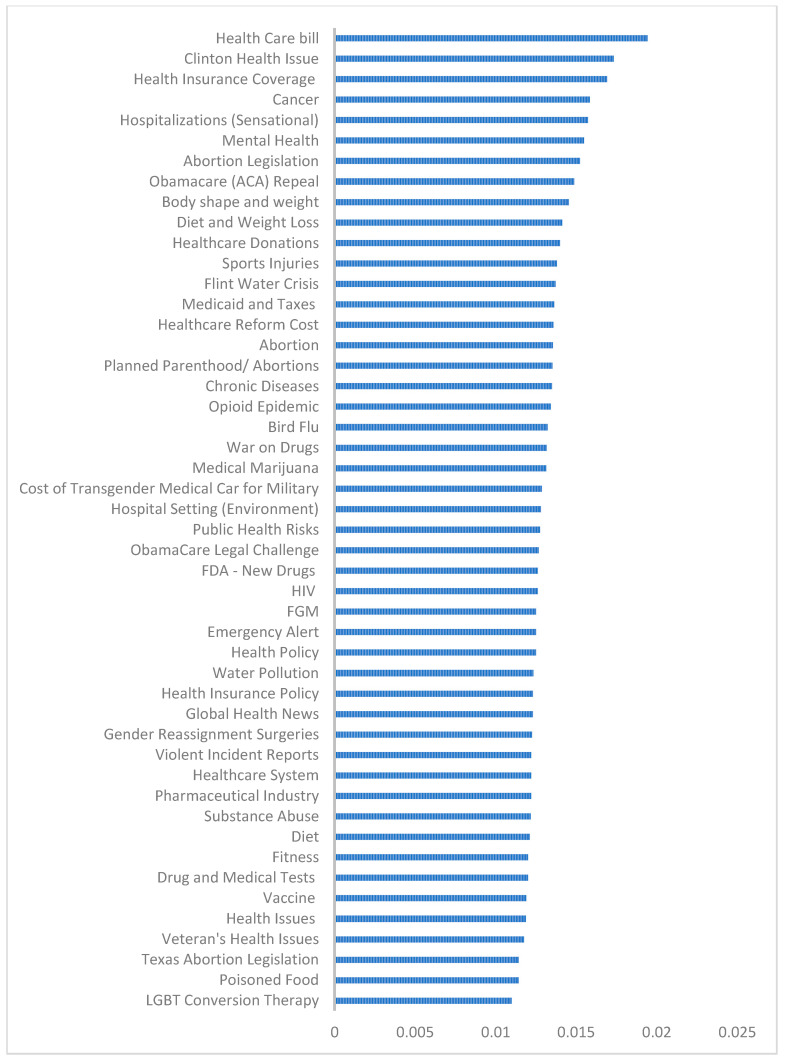
Overall weight of topics.

The 48 health topics are shown in Table 1 with a label representing the overall theme of the health topics. For example, the coders assigned “Clinton Health Issue” to Topic 2 containing the following words: “Hillary”, “Clinton”, “pneumonia”, “hillaryshealth”, “sick”, “records”, “medical”, “disease”, “lies”, and “parkinson”. Appendix A provides related tweets using P(T|D) offered by LDA for each of the 48 topics. We found that the trolls posted a wide range of topics such as abortion, opioids, and health policy. Figure 4 shows the overall weight of topics from the most discussed topic (healthcare bill) to the least discussed topic (LGBT conversion therapy).

We defined the passing *p*-value at 0.004 based on our sample size (15,249 tweets). Using the t-test and adjusting *p*-value with FDR implemented in R, the comparison of left (L) and right (R) Russian trolls based on the average weight of the 48 topics showed that there was not a significant difference (*p*-value > 0.004) between left and right Russian trolls on 31 (64.6%) topics (Table 2). However, there was a significant difference (*p*-value ≤ 0.004) between the left and right Russian trolls based on the average weight of 17 (35.4%) topics. Table 3 shows top 10 topics of left and right trolls based on the mean of each topic per tweet. The following findings are based on Figure 4 and Table 2 and Table 3.

Among the 17 topics, 10 topics were discussed more by left trolls than right ones (L > R) and seven topics were discussed more by right trolls than left ones (L < R).

While seven out of the top 10 topics were different for left and right trolls, there were three common topics discussed on both sides: health insurance coverage, healthcare bill, and cancer (Table 3).

Out of the overall top 10 topics in Figure 4, there was a significant difference between left and right trolls based on six topics: Healthcare Bill (L < R), Clinton Health Issue (L < R), Health Insurance Coverage (L > R), Hospitalizations (Sensational) (L > R), Mental Health (L > R), and Abortion Legislation (L < R).

Mental Health was the most popular topic among left trolls and was discussed significantly more than among right trolls (Table 2). This topic was also among the overall top 10 topics in Figure 4.

Clinton Health Issue was the most popular topic for right trolls (Table 3) and was discussed significantly more than for left trolls (Table 2). This topic was also among the overall top 10 topics in Figure 4.

Out of the top 10 topics of left trolls (Table 3), five topics were discussed more by left trolls than right trolls. Healthcare Bill was more popular for right trolls and cancer and the last three topics were discussed equally by both sides.

Out of the top 10 topics of right trolls (Table 3), five topics were discussed more by right trolls than left trolls. Health Insurance Coverage got more attention from left trolls than right ones and there was no significant difference between left and right trolls based on the average weight of Obamacare (ACA) Repeal, Cancer, and Diet and Weight Loss topics.

Two types of topics were discussed by the trolls. The first type was usually noncontroversial and nonpolitical topics (e.g., fitness). The second type was controversial and political topics (e.g., Obamacare).

## 4. Discussion

We aimed to identify and characterize the prevalence of health disinformation shared on social media by liberal and conservative Russian trolls on Twitter. The findings of this research suggest that Russian trolls discussed a variety of health topics ranging from diet and weight loss to vaccines. There were common health topics that have been important to American voters between 2016 and 2020, including healthcare, Medicaid, prescription drugs, health care laws, the opioid pandemic, Affordable Care Act (ACA), insurance, abortion, and the treatment of the LGBT community [59,60,61,62,63,64,65,66,67,68]. Russian trolls were not only talking about these important health issues but also have actively shared a wider range of health topics to shape public health opinion. This is an important finding, because previous work has shown that trolls promoted discord over just a few health topics such as vaccinations [9], but this work shows it is much broader.

The diversity of topics posted by both left and right trolls shows that Russian trolls generated health tweets for both left and right sides to promote polarized discussions. Out of 17 topics with a significant difference between left and right trolls, eight topics were discussed more by right trolls that support Donald Trump. This study confirms that the strategy of left trolls was to discuss different health issues that are of interests to both conservatives and liberals to divide the democratic party (e.g., discussing Clinton health issues) and promote health topics that are of interest to conservatives (e.g., abortion) [25]. In sum, the left and right trolls were politically polarized to divide America on topics specific to health, politically controversial topics related to health, as well the derision of Hillary Clinton’s health.

Previous studies have shown that identifying trolls is a difficult task, and human users have helped trolls spread false information unintentionally [69,70]. Findings from this paper emphasize that health disinformation is a global public health threat on social media for a considerable number of health topics. It is critical for health advocates to provide true health information to social media users and further educate them about false health information and promote online information literacy to the public. This study also helps social media sites identify and label a wider range of health topic information.

This study can be beneficial for researchers who are interested in health monitoring, communication, and promotion on social media by showing health information shared by Russian trolls. Our findings show that researchers need to expand their studies to include more health issues and develop new strategies for fighting misinformation. Our research also suggests that health agencies should work to develop new multiperspective policies to address the complicated strategies employed by trolls for dividing users. To combat false information, both social media companies and health agencies should also improve their social media activities and monitoring, address more health issues with clear and correct information, and develop strategies to increase the audience or number of followers.

While this study provides a new perspective on false health information, this research is not without limitations. First, we focused on trolls. It would be interesting to investigate spam and bots on social media. Second, we focused on the content of tweets. Studying patterns of using URLs in tweets and retweets could provide additional meaningful results.

## 5. Conclusions

Current relevant research focuses on limited health issues discussed by Russian trolls. This paper developed a method to identify health tweets shared by Russian trolls. Our findings show that trolls discussed 48 topics and there was a significant difference between left and right trolls on less than 50% of topics (e.g., abortion). However, there was not a significant difference between left and right trolls on majority of topics.

This study suggests new directions for research on health disinformation. Future research could address the limitations of this study by studying and comparing automated accounts (e.g., spams) and analyzing other features (e.g., the content of URLs in tweets). Future studies could also develop platforms to measure mental and financial impacts of trolls’ activities between 2012 and 2018.

## Figures and Tables

**Table 1:** Topics of tweets posted by Russian trolls.

ID	Label	Topic
T2	Clinton Health Issue	hillary clinton pneumonia hillaryshealth sick records medical disease lies parkinson
T3	Fitness	workout make exercise hard fitness day good stay summer body
T4	Health Issues	medicine health heart trust blood food find made wellness disease
T5	Health Policy	health public congress care trump job paying pays lose benefits
T6	Health Care bill	health care bill gop vote house trump plan senate republican
T9	Public Health Risks	world toxic health linked found food cancer epa air cancer-causing
T10	Abortion	abortion baby parts born body pro-life abortions women defends unborn
T12	Poisoned Food	kochfarms turkey happy poisoned thanksgiving omg launch friend usda sad
T13	Hospital Setting (Environment)	patients doctors work hospital cancer treatment nurses medical healthcare nhs
T14	Water Pollution	american water government falls poisoned live poison traitors native phosphorus
T15	Opioid Epidemic	drug opioid epidemic addiction heroin crisis deaths overdose dealer treat
T16	LGBT Conversion Therapy	therapy life pence mike year low lgbt wife changing conversion
T19	FDA—New Drugs	health news fda panel drug price human female backs secretary
T20	Healthcare System	health care system democrats republican traitor anti-trump warning approves
T21	Planned Parenthood/Abortions	planned parenthood control abortions birth form pitching pro-life responsible defund
T22	Flint Water Crisis	water lead flint poisoning toxic people children residents pay days
T23	Substance Abuse	alcohol pain people topl died drinking medication dangerous doctor told abuse
T25	Gender Reassignment Surgeries	life gender live nfl football set reassignment wins disgusting team
T27	ObamaCare Legal Challenge	court law health supreme obama case justice politics watch political
T28	Veteran’s Health Issues	veterans medical pill hospital receive immediately hours died vets private
T29	Medicaid and Taxes	medicaid tax cut trump medicare state social billion gop security
T30	Hospitalizations (Sensational)	hospital died man police family home woman year-old left injuries
T31	Emergency Alert	injured dead breaking emergency vastate declared kids illnesses visits listeria
T32	Diet and Weight Loss	health natural diet loss weight ways remove benefits top nutrition
T33	Body shape and weight	fat big ass body eat guy people weight lose obese
T34	Mental Health	mental health illness black issues suffering emotional depression stress anxiety
T35	Medical Marijuana	medical marijuana news cannabis legal oil treatment cigarette smoking weed
T36	Diet	diet eating food healthy unhealthy vegan meals junk alternatives sugar
T37	Health Insurance Policy	health major policy issues causing people die lack percent insurance
T39	Health Insurance Coverage	health care people insurance million americans plan coverage millions lose
T40	War on Drugs	drugs war drug johnson black problem marijuana death junkieus addicts
T42	Drug and Medical Tests	drug test high find school remember testing scientists passed discovered
T44	Bird Flu	news health flu local bird cases business officials deadly outbreak
T46	Healthcare Reform Cost	health care costs reform education universal end free concerns concerned
T47	Pharmaceutical Industry	drug big cost prices pharma companies lower americans industry prescription
T49	Violent Incident Reports	hospital secret report police officer reveals fire shot operating physically
T51	Female Genital Mutilation (FGM)	doctor female girls woman african genital black states american mutilation
T53	Healthcare Donations	money support million donate pay fund living medical children dollars
T56	HIV	hiv aids world infected cure lived days archived end drugs
T60	Chronic Diseases	disease risk heart diabetes obesity prevent researchers death attack chronic
T63	Vaccine	vaccine health diseases children call doctors critical spread harm effects
T64	Texas Abortion Legislation	takes texas step abortion breaking forward battling yuge moving stopping
T69	Sports Injuries	sports injury game left surgery live back baseball head nba
T72	Obamacare (ACA) Repeal	obamacare health insurance repeal healthcare congress trump gop support aca
T73	Global Health News	health news surgery south death released mers global fever science
T74	Cost of Transgender Medical Car for Military	heart surgery military transgender soldier plastic trump average medical wounded
T76	Abortion Legislation	abortion bill law ban passes women funding signs house pregnancy
T78	Cancer	cancer breast kills brain cells fight awareness cure survivor patients

**Table 2:** Comparison of left (L) and right (R) Trolls based on the mean of Health-related Topics (NS: *p*-value > 0.004; * *p*-value ≤ 0.004).

Topic	Results	Topic	Result
Clinton Health Issue	* L < R	Body shape and weight	NS
Fitness	* L > R	Mental Health	* L > R
Health Issues	NS	Medical Marijuana	NS
Health Policy	NS	Diet	NS
Health Care bill	* L < R	Health Insurance Policy	NS
Public Health Risks	NS	Health Insurance Coverage	* L > R
Abortion	* L < R	War on Drugs	* L > R
Poisoned Food	* L < R	Drug and Medical Tests	NS
Hospital Setting (Environment)	NS	Bird Flu	NS
Water Pollution	* L > R	Healthcare Reform Cost	NS
Opioid Epidemic	NS	Pharmaceutical Industry	NS
LGBT Conversion Therapy	* L > R	Violent Incident Reports	NS
FDA—New Drugs	NS	Female Genital mutilation (FGM)	* L > R
Healthcare System	* L < R	Healthcare Donations	NS
Planned Parenthood/Abortions	* L < R	HIV	NS
Flint Water Crisis	* L > R	Chronic Diseases	NS
Substance Abuse	NS	Vaccine	NS
Gender Reassignment Surgeries	NS	Texas Abortion Legislation	* L < R
ObamaCare Legal Challenge	NS	Sports Injuries	NS
Veteran’s Health Issues	NS	Obamacare (ACA) Repeal	NS
Medicaid and Taxes	NS	Global Health News	NS
Hospitalizations (Sensational)	* L > R	Cost of Trans gender Medical Car for Military	NS
Emergency Alert	* L < R	Abortion Legislation	NS
Diet and Weight Loss	NS	Cancer	NS

**Table 3:** Top 10 topics of left and right trolls based on the average weight of each topic per tweet. The three common topics are shown in bold-italic format. Topics that are underlined are also among the overall top 10 topics in Figure 4.

Left Trolls	Right Trolls
Mental Health (L > R)	Clinton Health Issue (L < R)
***Health Insurance Coverage (L > R)***	***Health Care bill (L < R)***
Hospitalizations (Sensational) (L > R)	Planned Parenthood/Abortions (L < R)
Flint Water Crisis (L > R)	Abortion Legislation (L = R)
***Health Care bill (L < R)***	Obamacare (ACA) Repeal (L = R)
***Cancer (L = R)***	Abortion (L < R)
War on Drugs (L > R)	***Cancer (L = R)***
Sports Injuries (L = R)	***Health Insurance Coverage (L > R)***
Body shape and weight (L = R)	Diet and Weight Loss (L = R)
Medicaid and Taxes (L = R)	Emergency Alert (L < R)

## Data Availability

Not applicable.

## Appendix A

**Table A1:** An example of tweets for each topic.

ID	Label	An Example of Tweets
T2	Clinton Health Issue	*Well looks like Hillary’s pneumonia caused by Parkinsons disease*
T3	Fitness	*Shoulder/Abdominal workouts have been brutal this month..progress..quotes quote gym gymlife*
T4	Health Issues	*Have some dark chocolate (an ounce/dayindulgecan lower blood pressureincrease blood flow improve good cholesterol (HDL)*
T5	Health Policy	*Trump Wants to Know Why Congress Isnt Paying What the Public Pays for Health Care*
T6	Health Care bill	*Constituents applaud GOP senator for opposing health care bill*
T9	Public Health Risks	*Pepsi admits its soda contains cancer-causing ingredients cancer soda pepsi diet*
T10	Abortion	*Abortion Clinic Killed This Woman in a Botched Abortion. Then Her Organs Were Harvested*
T12	Poisoned Food	*Happy Thanksgiving Turkey was poisoned? Turkey Food Poisoning USDA*
T13	Hospital Setting (Environment)	*local Desperate patients search for Minnesota doctors who will help them sign up for medical marijuana*
T14	Water Pollution	*Water in American Falls is poisoned with phosphorus waste I suppose that our government wants to poison us phosphorus disaster Traitors*
T15	Opioid Epidemic	*Seattle Approves Safe Injection Sites for Illegal Drug Users to Inject Illegal Drugs*
T16	LGBT Conversion Therapy	*So much so that they can disregard gay conversion therapy supporter Mike Pence*
T19	FDA and New Drugs	*news FDA panel backs female sex pill under safety conditions*
T20	Healthcare System	*The largest example of government-run health care in the U.S should be a warning to all #WakeUpAmerica*
T21	Planned Parenthood/Abortions	*Planned Parenthood U.KNow Pitching Abortions as Just a Form of Birth Control barr*
T22	Flint Water Crisis	*They’re pumping millions of gallons of water for free while flint residents pay for poisoned water.*
T23	Substance Abuse	*the many people who must use pain killers temporarily they’re a God send but just like alcohol abuse.*
T25	Gender Reassignment Surgeries	*Gender Reassignment Surgery Has Just Reached Disgusting New Heights*
T27	ObamaCare Legal Challenge	*Obama defends health care law ahead of Supreme Court ruling*
T28	Veteran’s Health Issues	*Veterans at Wisconsin VA Hospital May Have Been Infected with Hepatitis HIV*
T29	Medicaid and Taxes	*Trump is backing a bill that cuts Medicaid by nearly a trillion dollars. It also gives a big tax cut to the rich.*
T30	Hospitalizations (Sensational)	*Hospitalized woman deprived of water died in jail after arrest for unpaid fines BLM*
T31	Emergency Alert	*BREAKING One Dead Injured in #VAState of Emergency Declared!*
T32	Diet and Weight Loss	*What weight loss drink did Sherman use on #NuttyProfessor? Jenny Craig, Mega Shake, Super Diet, Weight Off*
T33	Body shape and weight	*This is what lbs of muscle* vs. *lbs of fat look Focus on what youre made of not your weight Daily Reminder*
T34	Mental Health	*I learned how to prioritize my mental health. That depression wasn’t my fault. That anxiety wasn’t my fault. That there was no shame.*
T35	Medical Marijuana	*Medical marijuana isn’t enough. It’s not legal until it’s legal for black and brown communities who are still x more likely to be arrested!*
T36	Diet	*If taking junk food out of my diet improves my health taking junk media out of my life improves my soul quote*
T37	Health Insurance Policy	*Trying to figure out how #TakeAKnee is un-American but letting people die because of lack of health insurance is patriotic #TrumpBudget*
T39	Health Insurance Coverage	*Throwing million people off their health care to give billionaires a tax break is heartless & amp irresponsible. We cannot pass #Trumpcare.*
T40	War on Drugs	*Nixon Official Admits The War on Drugs Was Really A War On Blacks And It Worked.*
T42	Drug and Medical Tests	*NC High Schooler Who Passed Drug Test Suspended for Smelling Like Weed #BlackTwitter*
T44	Bird Flu	*Growth in Iowa bird flu cases appears to be slowing health*
T46	Healthcare Reform Cost	*Despite Obama Promise, sHealth Care Costs Have Risen the Most in Years Breitbart*
T47	Pharmaceutical Industry	*When big pharma jacks up the prices of a life-saving medicationthe cost isn’t just dollars and cents--it’s livesYeson*
T49	Violent Incident Reports	*top RT @AnnaBDAlgerian Paris Attacker Identified in Hospital After Being Shot Five Times During Arrest*
T51	Female genital mutilation (FGM)	*Detroit doctor arrested for performing Female Genital Mutilation on girls ages six to eight years old. Feminists are silent.*
T53	Healthcare Donations	*Please Support Help the Dave Fredrick Family with medical expenses Donate.*
T56	HIV	*EXCLUSIVE:Clinton Foundation AIDS Program Distributed Watered-Down Drugs To Third World Countries.*
T60	Chronic Diseases	*Johnson Johnson starts project to prevent Type diabetes health*
T63	Vaccine	*VaccinateUS vaccines eradicated smallpox and have nearly eradicated other diseases such as polio.*
T64	Texas Abortion Legislation	*BREAKING Texas Takes a YUGE Step Forward in Battling Abortion amelin*
T69	Sports Injuries	*sports Cavs say Kyrie Irving has fractured left knee cap will have surgery and miss months*
T72	Obamacare (ACA) Repeal	*This is rich House GOP exempt themselves their health insurance from their latest ACA repeal plan*
T73	Global Health News	*South Korea reports sixth death from Middle East Respiratory Syndrome as government steps up monitoring*
T74	Cost of Transgender Medical Car for Military	*Transgender Medical Care SHOCKINGLY More Costly than Average Military Soldier.*
T76	Abortion Legislation	*Top News Florida governor signs bill requiring two clinic visits waiting period for abortion*
T78	Cancer	*Cancer Killing Molecule Destroys Tumor Cells and Leaves Normal Cells Unaffected*
